# Risk score to predict false-positive ST-segment elevation myocardial infarction in the emergency department: a retrospective analysis

**DOI:** 10.1186/s13049-017-0408-7

**Published:** 2017-06-30

**Authors:** Ji Hoon Kim, Yun Ho Roh, Yoo Seok Park, Joon Min Park, Bo Young Joung, In Cheol Park, Sung Phil Chung, Min Joung Kim

**Affiliations:** 1 Department of Emergency Medicine, Yonsei University College of Medicine, 50 Yonsei-ro, Seodaemun-gu, 03722 Seoul, Republic of Korea; 2 Department of Research Affairs, Biostatistics Collaboration Unit, Yonsei University College of Medicine, 50 Yonsei-ro, Seodaemun-gu, 03722 Seoul, Republic of Korea; 3 Department Emergency Medicine, Inje University Ilsan Paik Hospital, 170 Juhwa-ro, Ilsanseo-gu, 10380 Goyang-si, Gyeonggi-do Republic of Korea; 4 Division of Cardiology, Department of Internal medicine, Yonsei University College of Medicine, 50 Yonsei-ro, Seodaemun-gu, 03722 Seoul, Republic of Korea; 5 Department of Emergency Medicine, Severance Hospital, 50 Yonsei-ro, Seodaemun-gu, Seoul, 120-752 Republic of Korea

**Keywords:** ST-segment elevation myocardial infarction, Percutaneous coronary intervention, Electrocardiography, Risk score, Predictive model

## Abstract

**Background:**

The best treatment approach for ST-segment elevation myocardial infarction (STEMI) is prompt primary percutaneous coronary intervention (PCI). However, some patients show ST elevation on electrocardiography (ECG), but do not have myocardial infarction. We sought to identify the frequency of and to develop a prediction model for false-positive STEMI.

**Methods:**

This study was conducted in the emergency departments (EDs) of two hospitals using the same critical pathway (CP) protocol to treat STEMI patients with primary PCI. The prediction model was developed in a derivation cohort and validated in internal and external validation cohorts.

**Results:**

Of the CP-activated patients, those for whom ST elevation did not meet the ECG criteria were excluded. Among the patients with appropriate ECG patterns, the incidence of false-positive STEMI in the entire cohort was 16.3%. Independent predictors extracted from the derivation cohort for false-positive STEMI were age < 65 years (odds ratio [OR], 2.54; 95% confidence interval [CI], 1.35–4.89), no chest pain (OR, 12.04; 95% CI, 5.92–25.63), atypical chest pain (OR, 7.40; 95% CI, 3.27–17.14), no reciprocal change (OR, 4.80; 95% CI, 2.54–9.51), and concave-morphology ST elevation (OR, 14.54; 95% CI, 6.87–34.37). Based on the regression coefficients, we established a simplified risk score. In the internal and external validation cohorts, the areas under the receiver operating characteristic curves for our risk score were 0.839 (95% CI, 0.724–0.954) and 0.820 (95% CI, 0.727–0.913), respectively; the positive predictive values were 40.9% and 22.0%, respectively; and the negative predictive values were 94.9% and 96.7%, respectively.

**Discussion:**

Our prediction model would help them make rapid decisions with better rationale.

**Conclusion:**

We devised a model to predict false-positive STEMI. Larger-scale validation studies are needed to validate our model, and a prospective study to determine whether this model is effective in reducing improper primary PCI in actual clinical practice should be performed.

## Background

Primary percutaneous coronary intervention (PCI) is a standardized treatment approach for ST-segment elevation myocardial infarction (STEMI). Timely reperfusion therapy is especially crucial for salvaging reversible infarcted lesions and minimizing myocardial damage. Therefore, the American College of Cardiology/American Heart Association (ACC/AHA) guidelines recommend that therapy be conducted within 90 min of door-to-balloon time [1]. Multidisciplinary cooperation is necessary to achieve this target time; therefore, many emergency departments (EDs) use the critical pathway (CP) protocol [2, 3]. The protocol provides a guide for each department to simplify the decision-making process and enable prompt action. The door-to-balloon time for STEMI patients has been shortened in the past several years [4–6]. However, some patients show ST elevation on electrocardiography (ECG), but do not have myocardial infarction (MI). This is called false-positive STEMI. As the door-to-balloon time of STEMI patients has been reduced, the number of patients who are falsely diagnosed with STEMI in whom unnecessary emergency coronary angiography (CAG) is performed has increased [7].

Several studies have been conducted to date on false-positive STEMI patients. Emergency CAG performed in patients with false-positive STEMI is not uncommon, with reported rates varying between 7.5% and 36.0% [8–14]. CAG is an invasive procedure that increases risks for patients and medical expenditures. The procedure can affect patient safety because other tests and treatments are delayed, and the diagnosis cannot be made in a timely manner [7, 15]. Recently, several attempts have been made to reduce unnecessary CAG by investigating predictors of falsely diagnosing a patient with STEMI [8, 9, 16]. One study has suggested the use of a prediction model for false-positive STEMI [16]. However, the decision about whether to perform emergency procedures in patients with suspected STEMI still depends on the competence of individual physicians, and the false diagnosis of STEMI continues to occur.

The objectives of our study were to determine the frequency of false-positive STEMI diagnosis in our system, to develop a prediction model for false-positive STEMI, and to validate this model internally and externally.

## Methods

### Study setting and participants

This was a retrospective, observational study of prospectively collected data in two urban tertiary teaching hospitals. The derivation and internal validation cohorts were derived from hospital A, and the external validation cohort was obtained from hospital B. Both hospitals are located in Seoul, the capital city of Korea, and both have level 2 EDs. Hospital A is located in the northwest of the city, which is responsible for treating emergency patients from three districts with a population of 1.13 million, and 80,000 patients visit the ED every year. Hospital B, which is an affiliated hospital of A, is located in the southeast, covering two districts comprising 920,000 people, with 50,000 annual ED visits. The two hospitals have 24 and 7 catheterization rooms, respectively, and perform approximately 4000 and 1500 CAGs per year, respectively. Our research was approved by each hospital’s institutional review board, and patient consent was waived owing to the retrospective nature of the study. The methodological quality was assessed by 3 authors (M.J. Kim, Y.H. Roh, and Y.S. Park) using the QUADAS criteria [17].

Patients who were examined between January 2010 and December 2013 in the ED of hospital A were randomly divided into the derivation (75%) and internal validation (25%) cohorts. Data on the external validation cohort were obtained between January 2010 and December 2012 from hospital B. In both hospitals, the same CP protocol was used for patients with STEMI; when STEMI was suspected based on clinical symptoms and ECG findings, the emergency physician activated the CP. Then, the ED nurse, diagnostic laboratory, cardiologist, catheterization room staff, and transport staff were mobilized, and the cardiologist performed primary PCI. We investigated patients older than 18 years who underwent the CP in the derivation, internal validation, and external validation cohorts. The CP protocol included new-onset left bundle branch block (LBBB) as well as ST-segment elevation. Our inclusion criterion was the only CP activation for patients with ST-segment elevation. In some patients, the CP was activated based on ST-segment elevation, but ST elevation was not clear on ECG. The exclusion criterion was no definite ST-segment elevation on ECG.

### Data collection and definitions

We determined ST-segment elevation according to the criteria in the ACC/AHA guideline. The criteria are a J-point elevation in two or more contiguous leads with a cut-off value of ≥0.1 mV (1 mm) in all leads other than V2 and V3, for which the following cut-off values were applied: 0.2 mV (2 mm) in men ≥40 years old, 0.25 mV (2.5 mm) in men <40 years old, or 0.15 mV (1.5 mm) in women [18].

False-positive STEMI was defined as a lack of a culprit artery observed on CAG. A culprit lesion was identified on CAG if there was total or subtotal occlusion or stenosis >70% (>50% in the left main coronary artery) with a visible thrombus or other features that suggested acute plaque rupture in the coronary artery corresponding to ST-segment elevation on ECG. However, some patients did not undergo CAG for various reasons, such as an absence of consent to undergo the procedure, uncertainty of the clinical benefit for patients with terminal illness, or those with cardiac arrest prior to coronary intervention. We applied a clinical scenario to determine whether these patients had false-positive STEMI. The clinical scenario was: 1) the patient was diagnosed with another disease in which the ECG finding was clearly explicable before discharge; and 2) the patient was not diagnosed with another disease, but did not show elevated levels of cardiac biomarkers or receive any treatments for myocardial infarction (MI) during hospitalization. The first biomarker assay result was determined as positive when the troponin I value was ≥0.2 ng/mL (reference interval: <0.2 ng/mL) or the creatine kinase-MB value was ≥7% of the creatine kinase value.

We extracted data from the patients’ electronic medical records that included ECG and angiographic results. The patients’ baseline characteristics, underlying disease, cardiovascular risk factors, typical chest pain, onset time of symptoms, radiating pain, method of ED arrival, hemodynamic instability in the ED, and ECG patterns were investigated. Each ECG lead was reviewed for the presence of ST elevation, height and shape of ST elevation, and the presence of reciprocal changes. According to the involved leads, the ST elevation locations were classified as anterior, inferior, posterolateral, or diffuse. The anterior area was defined as an ST elevation in two or more adjacent leads among V1 to V4. The inferior area was defined as an ST-segment elevation in two or more adjacent leads among II, III, and aVF. The lateral area was defined as an ST-segment elevation in two or more adjacent leads among I, aVL, V5, and V6. The posterior area was defined as an ST-segment of 0.05 mV or greater in leads V7 to V9 but 0.1 mV or greater in men younger than 40 years. Posterolateral wall MI was defined as ST-segment elevation in two or more adjacent leads near the posterior and lateral walls. A diffuse location of ST elevation referred to ST elevation that was distributed in more than one coronary artery area. We analysed the maximal height of the ST elevation, which was the greatest height in all leads with ST elevation. For the shape of the ST elevation, we determined whether the morphology was concave based on the line from the J point to the end of the ST segment [10]. ST depression was recognized as a reciprocal change if it was in the anterior or lateral areas for inferior ST elevation, in the inferior area for anterior or lateral ST elevation, or in the anterior area for posterior ST elevation [19]. We also checked for the presence of a Q wave and left ventricular hypertrophy. Left ventricular hypertrophy was defined using an ECG computer algorithm.

### Statistical analysis

We built a predictive model for false-positive STEMI using data from the derivation cohort. A univariate analysis was performed using independent t-tests for continuous variables and χ^2^ tests for categorical variables. To determine the independent predictors for false-positive STEMI, a multivariable logistic regression analysis was conducted. The determination of clinically significant factors was based on previous studies and factors associated with false-positive STEMI on the univariate analysis (*p* < 0.1). Logistic regression coefficients were used to generate a risk score for false-positive STEMI. To facilitate clinical application of this score, the coefficient of each variable was divided by the lowest beta values, multiplied by a constant, and rounded to the nearest integer. In the derivation dataset, calibration was assessed using the Hosmer-Lemeshow goodness-of-fit test for the simplified score model. The final risk score was validated in the internal and external validation cohorts. The predictability of the risk score for false-positive STEMI was assessed by calculating the area under the receiver operating characteristic (AUROC) curve. We employed three cohorts to test the diagnostic characteristics of the cut-off point for the final model based on standard validation measures: sensitivity, specificity, positive predictive value (PPV), and negative predictive value (NPV). The cut-off value was chosen for our predictive model to maximize the Youden Index (defined as sensitivity + specificity of 1) [20]. All *p*-values were two-sided, and 95% confidence intervals (CI) were calculated for odds ratios (ORs). All analyses were performed using SAS version 9.4 (SAS Institute, Cary, NC, USA) and R version 3.3.2 (The R Foundation for Statistical Computing, Vienna, Austria).

## Results

### Cohort analysis

**Fig. 1:**
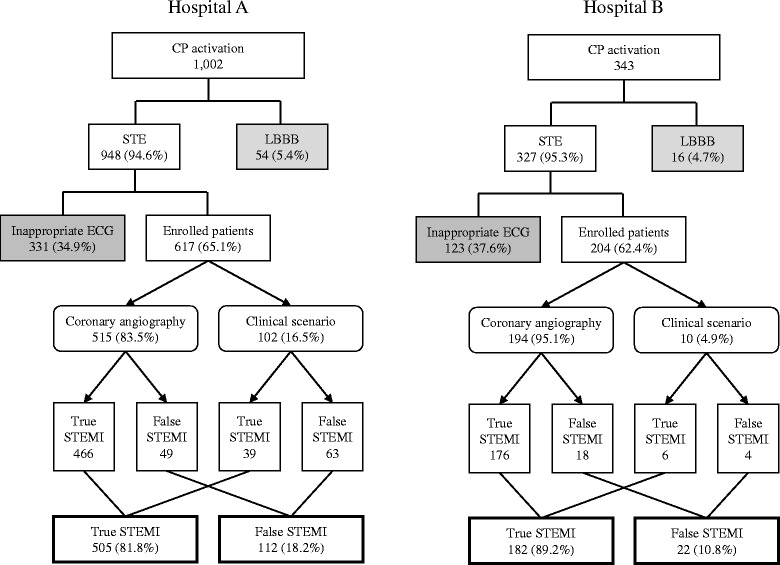
Flow chart showing the critical pathway activation protocol and study enrolment. The derivation and internal validation cohorts were extracted from hospital A; the external validation cohort was extracted from hospital B. CP, critical pathway; STE, ST elevation; LBBB, left bundle branch block; ECG, electrocardiography; STEMI, ST-segment elevation myocardial infarction

In the derivation and internal validation cohorts (hospital A), the CP was activated in 1002 patients during the study period (Fig. 1). Nine patients with ECG loss and two patients who died before further evaluation were excluded. The CP was activated due to new-onset LBBB in 54 (5.4%) patients. Of the 948 (94.6%) patients whose protocol was activated by ST elevation, 331 (34.9%) did not meet the criteria for ST-segment elevation. Although the ST-segment elevation was not clear, 77 (23.3%) patients underwent emergent CAG, and 23 patients were diagnosed with non-STEMI (NSTEMI). Among the 331 patients, 109 (32.9%) patients with NSTEMI, including 86 patients who were not treated with primary PCI but were diagnosed with NSTEMI, followed by no diagnosis (43, 13.1%), structural/valvular heart disease (37, 11.1%), and primary rhythm disturbance (35, 10.6%). Finally, 617 patients with appropriate ST-segment elevation were included in the study. We applied a clinical scenario to determine false-positive STEMI in 102 (16.5%) patients who did not undergo primary PCI. The overall frequency of false-positive STEMI was 112 (18.2%). In the external validation cohort (hospital B), 204 patients were included, and 22 (10.8%) patients were falsely diagnosed with STEMI. The overall incidence of false-positive STEMI for both hospitals was 16.3%.

The baseline characteristics of the derivation and the internal and external validation cohorts are shown in Table 1. The external validation cohort had a greater proportion of patients who were younger than 65 years of age compared with those in the derivation cohort (51.8% vs. 63.7%, *p* = 0.004), and more patients smoked (45.8% vs. 56.9%, *p* = 0.008). There were more cases of ambulance arrival (46.0% vs. 54.4%, *p* = 0.042), and fewer Q waves were observed on ECG (4.3% vs. 0.5%, *p* = 0.007) in the external validation cohort compared with that in the derivation cohort. The frequency of false-positive STEMI was also lower in the external validation cohort (18.2% vs. 10.8%, *p* = 0.015) than in the derivation cohort.

**Table 1:** Characteristics of the patients in the derivation and internal/external validation cohorts

	Derivation (*n* = 494)	Internal validation (*n* = 123)	P1-value	External validation (*n* = 204)	P2-value
Age < 65 years	256 (51.8)	68 (55.3)	0.491	130 (63.7)	0.004
Male, n (%)	400 (81.0)	93 (75.6)	0.184	166 (81.4)	0.902
BMI, mean ± SD	23.7 mea	23.9 mea	0.509	24.99mea	<0.001
Diabetes mellitus, n (%)	162 (32.8)	34 (27.6)	0.272	65 (31.9)	0.811
Hypertension, n (%)	256 (51.8)	69 (56.1)	0.395	101 (49.5)	0.578
Chronic renal failure, n (%)	23 (4.7)	7 (5.7)	0.633	6 (2.9)	0.302
Hypercholesterolemia, n (%)	68 (13.8)	14 (11.4)	0.486	38 (18.6)	0.104
Current smoking, n (%)	226 (45.8)	49 (39.8)	0.238	116 (56.9)	0.008
Previous event, n (%)					
Stroke	21 (4.3)	9 (7.3)	0.157	7 (3.4)	0.616
Variant angina	7 (1.4)	1 (0.8)	1.000	2 (1.0)	1.000
Unstable angina	30 (6.1)	7 (5.7)	0.873	7 (3.4)	0.157
Myocardial infarction	45 (9.1)	8 (6.5)	0.356	6 (2.9)	0.004
CAD	82 (16.6)	13 (10.6)	0.097	16 (7.8)	0.002
Heart failure	16 (3.2)	0	0.052	3 (1.5)	0.305
Previous PCI	73 (14.8)	15 (12.2)	0.464	16 (7.8)	0.012
Previous CABG	11 (2.2)	1 (0.8)	0.476	1 (0.5)	0.196
Family history of CAD, n (%)	31 (6.3)	5 (4.1)	0.518	16 (7.8)	0.452
Chest pain, n (%)					
Typical	350 (70.9)	84 (68.3)	0.850	151 (74.0)	0.175
Atypical	57 (11.5)	15 (12.2)		14 (6.9)	
No chest pain	87 (17.6)	24 (19.5)		39 (19.1)	
Other symptom, n (%)	215 (43.5)	48 (39.0)	0.367	65 (31.9)	0.004
Radiating pain, n (%)	139 (28.1)	27 (22.0)	0.166	59 (28.9)	0.834
Symptom onset to arrival, n (%)					
≤ 6 h	383 (77.5)	79 (64.2)	0.003	169 (82.8)	0.064
6-24 h	63 (12.8)	30 (24.4)		26 (12.7)	
> 24 h	48 (9.7)	14 (11.4)		9 (4.4)	
Ambulance arrival, n (%)	227 (46.0)	40 (32.5)	0.007	111 (54.4)	0.042
Hemodynamic instability, n (%)	88 (17.8)	20 (16.3)	0.685	35 (17.2)	0.836
Positive baseline biomarker, n (%)	199 (40.3)	58 (47.2)	0.167	63 (30.9)	0.020
Location of STE, n (%)					
Anterior	209 (42.3)	57 (46.3)	0.253	82 (40.2)	0.474
Inferior	190 (38.5)	40 (32.5)		75 (36.8)	
Posterolateral	19 (3.8)	9 (7.3)		6 (2.9)	
Diffuse	76 (15.4)	17 (13.8)		41 (20.1)	
Height of maximal STE (mm)	3.1±2.0	3.2 ± 2.1	0.581	3.5 ± 2.1	0.049
Number of leads with STE	3.2 ± 1.2	3.1 ± 1.1	0.505	3.5 ± 1.3	0.007
No reciprocal change, n (%)	252 (51.0)	62 (50.4)	0.904	126 (61.8)	0.010
Concave morphology of STE, n (%)	243 (49.2)	51 (41.5)	0.125	94 (46.1)	0.454
Q wave, n (%)	21 (4.3)	4 (3.3)	0.800	1 (0.5)	0.007
LVH, n (%)	70 (14.2)	14 (11.4)	0.420	25 (12.3)	0.502
False-positive STEMI, n (%)	90 (18.2)	22 (17.9)	0.932	22 (10.8)	0.015

P1 denotes the *P*-value that compares the derivation and internal validation cohorts, and P2 denotes the *P*-value that compares the derivation and external validation cohorts. *SD* standard deviation, *BMI* body mass index, *CAD*coronary artery disease, *PCI* percutaneous coronary intervention, *CABG* coronary artery bypass graft, *STE* ST elevation, *LVH* left ventricular hypertrophy, *STEMI* ST-segment elevation myocardial infarction, *SD* standard deviation

### Model development

The clinical characteristics of the STEMI and false-positive STEMI patients were compared in the derivation cohort (Table 2). In the univariate analysis, patients who were falsely diagnosed with STEMI were younger than the STEMI patients (age < 65 years, 47.3% vs. 72.2%, *p* < 0.001) and were more likely to be male (79.2% vs. 88.9%, *p* = 0.038). The prevalence of diabetes mellitus, hypertension, and hypercholesterolemia was higher in patients with STEMI than in falsely diagnosed patients, but a history of variant angina was more common in false-positive STEMI patients (0.5% vs. 5.6%, *p* = 0.004) than in STEMI patients. More patients with STEMI complained of typical chest pain (78.7% vs. 35.6%, *p* < 0.001) and radiating pain (30.9% vs. 15.6%, *p* = 0.004) than did false-positive STEMI patients. Positive baseline biomarkers were observed more often in STEMI than in false-positive STEMI patients (45.5% vs. 16.7%, *p* < 0.001). The ST-segment elevation in false-positive STEMI patients tended to be located in the anterior area compared with that in STEMI patients (38.4% vs. 60.6%, *p* < 0.001). In addition, the height of the ST elevation was lower and the number of leads with ST elevation was less in false-positive STEMI patients than in STEMI patients. Reciprocal change was more common in STEMI patients than in false-positive STEMI patients (44.8% vs. 78.9%, *p* < 0.001). More patients with falsely diagnosed STEMI had concave ST elevation shapes than did STEMI patients (40.1% vs. 90.0%, *p* < 0.001).

**Table 2:** Comparison between the STEMI and false-positive STEMI patients in the derivation cohort

	STEMI (*n* = 404)	False-positive STEMI (*n* = 90)	OR (95% CI)	*P*-value
Age < 65 years	191 (47.3)	65 (72.2)	2.90 (1.76–4.79)	<0.001
Male, n (%)	320 (79.2)	80 (88.9)	2.10 (1.04–4.23)	0.038
BMI, mean ± SD	23.8 ± 3.6	23.1 ± 3.5	0.95 (0.89–1.01)	0.121
Diabetes mellitus, n (%)	142 (35.1)	20 (22.2)	0.53 (0.31–0.90)	0.020
Hypertension, n (%)	220 (54.5)	36 (40.0)	0.56 (0.35–0.89)	0.014
Chronic renal failure, n (%)	17 (4.2)	6 (6.7)	1.63 (0.62–4.25)	0.321
Hypercholesterolemia, n (%)	63 (15.6)	5 (5.6)	0.32 (0.12–0.82)	0.017
Current smoking, n (%)	193 (47.8)	33 (36.7)	0.63 (0.40–1.01)	0.057
Previous event, n (%)				
Stroke	20 (5.0)	1 (1.1)	0.22 (0.03–1.63)	0.137
Variant angina	2 (0.5)	5 (5.6)	11.82 (2.26–61.93)	0.004
Unstable angina	25 (6.2)	5 (5.6)	0.89 (0.33–2.40)	0.820
Myocardial infarction	37 (9.2)	8 (8.9)	0.97 (0.44–2.16)	0.937
CAD	68 (16.8)	14 (15.6)	0.91 (0.49–1.70)	0.769
Heart failure	13 (3.2)	3 (3.3)	1.04 (0.29–3.72)	0.955
Previous PCI	60 (14.9)	13 (14.4)	0.97 (0.51–1.85)	0.922
Previous CABG	9 (2.2)	2 (2.2)	1.00 (0.21–4.70)	0.998
Family history of CAD, n (%)	29 (7.2)	2 (2.2)	0.29 (0.07–1.26)	0.098
Chest pain, n (%)				
Typical	318 (78.7)	32 (35.6)	1 (reference)	<0.001
Atypical	37 (9.2)	20 (22.2)	5.37 (2.79–10.33)	
No chest pain	49 (12.1)	38 (42.2)	7.71 (4.41–13.47)	
Other symptom, n (%)	160 (39.6)	55 (61.1)	2.40 (1.50–3.83)	<0.001
Radiating pain, n (%)	125 (30.9)	14 (15.6)	0.41 (0.22–0.76)	0.004
Symptom onset to arrival, n (%)				
≤ 6 h	319 (79.0)	64 (71.1)	1 (reference)	0.186
6-24 h	50 (12.4)	13 (14.4)	1.30 (0.67–2.52)	
> 24 h	35 (8.7)	13 (14.4)	1.85 (0.93–3.69)	
Ambulance arrival, n (%)	181 (44.8)	46 (51.1)	1.29 (0.82–2.04)	0.278
Hemodynamic instability, n (%)	73 (18.1)	15 (16.7)	0.91 (0.49–1.67)	0.753
Positive baseline biomarker, n (%)	184 (45.5)	15 (16.7)	0.24 (0.13–0.43)	<0.001
Location of STE, n (%)				
Anterior	155 (38.4)	54 (60.6)	1 (reference)	
Inferior	165 (40.8)	25 (27.8)	0.44 (0.26–0.73)	0.002
Posterolateral	14 (3.5)	5 (5.6)	1.03 (0.35–2.98)	0.964
Diffuse	70 (17.3)	6 (6.7)	0.25 (0.10–0.60)	0.002
Height of maximal STE (mm)	3.3 ± 2.1	2.4 ± 1.1	0.72 (0.61–0.85)	<0.001
Number of leads with STE	3.3 ± 1.2	2.8 ± 1.0	0.61 (0.47–0.81)	<0.001
No reciprocal change, n (%)	181 (44.8)	71 (78.9)	4.60 (2.68–7.92)	<0.001
Concave morphology of STE, n (%)	162 (40.1)	81 (90.0)	13.4 (6.56–27.53)	<0.001
Q wave, n (%)	21 (5.2)	0	<0.01 (<0.01- > 99.99)	0.971
LVH, n (%)	52 (12.9)	18 (20.0)	1.69 (0.94–3.06)	0.082

*STEMI* ST-segment elevation myocardial infarction, *OR* odds ratio, *CI* confidence interval, *SD* standard deviation, *BMI* body mass index, *CAD* coronary artery disease, *PCI* percutaneous coronary intervention, *CABG* coronary artery bypass graft, *STE* ST elevation, *LVH* left ventricular hypertrophy

To identify predictors for false-positive STEMI, a multivariable logistic regression analysis was performed (Table 3). The independent predictors were age < 65 years (OR, 2.54; 95% CI, 1.35–4.89; *p* = 0.004), absence of chest pain (OR, 12.04; 95% CI, 5.92–25.63; *p* < 0.001) or atypical chest pain (OR, 7.40; 95% CI, 3.27–17.14; *p* < 0.001), no reciprocal change (OR, 4.80; 95% CI, 2.54–9.51; *p* < 0.001), and concave-morphology ST elevation (OR, 14.54; 95% CI, 6.87–34.37; *p* < 0.001). Based on the regression coefficients, we established a risk score system to predict false-positive STEMI by assigning a simplified score to these factors (Table 3). The range of the total risk score was between 0 and 8 points. The *p-*value for the Hosmer-Lemeshow test was 0.574, suggesting that our model was well calibrated.

**Table 3:** Multivariable predictors of false-positive STEMI in the derivation cohort and simplified risk score

Variables	Beta	OR (95% CI)	*P* value	Risk score
Age				
≥ 65 years		1 (reference)		0
< 65 years	0.93	2.54 (1.35–4.89)	0.004	1
Chest pain				
Typical		1 (reference)		0
Atypical	2.00	7.40 (3.27–17.14)	<0.001	2
No	2.49	12.04 (5.92–25.63)	<0.001	2.5
Reciprocal change				
Yes		1 (reference)		0
No	1.57	4.80 (2.54–9.51)	<0.001	1.5
Concave morphology of STE				
Yes	2.68	14.54 (6.87–34.37)	<0.001	3
No		1 (reference)		0

*STEMI* ST-segment elevation myocardial infarction, *OR* odds ratio, *CI* confidence interval, *STE* ST elevation

### Validation of the model

**Fig. 2:**
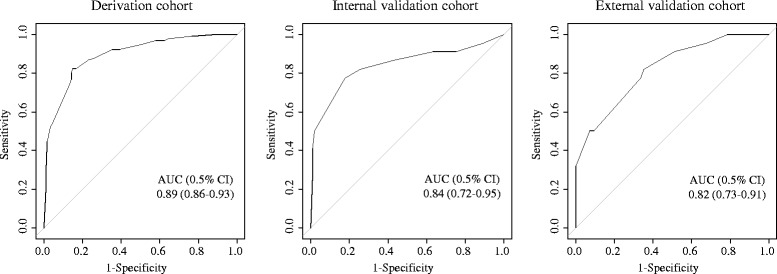
Risk scores for false-positive STEMI in the derivation, internal validation, and external validation cohorts. Receiver operating characteristic curves for the risk scores for false-positive ST-segment elevation myocardial infarction in the derivation, internal validation, and external validation cohorts; AUC, area under the curve; CI, confidence interval; STEMI, ST-segment elevation myocardial infarction

The ability of our final model to predict false-positive STEMI in the derivation cohort was 0.893 (95% CI 0.856–0.930), as estimated by the AUROC curve (Fig. 2). In the internal and external validation cohorts, the simplified risk score showed good discrimination for false-positive STEMI, with AUROCs of 0.839 (95% CI 0.724–0.954) and 0.820 (95% CI 0.727–0.913), respectively. Based on the Youden Index, a cut-off point of ≥2.5 was used to predict false-positive STEMI in the derivation cohort. Based on this cut-off value, the sensitivity and specificity of false-positive STEMI were 83.3% and 76.2%, respectively, the PPV was 43.9%, and the NPV was 93.4% (Table 4). In the internal and external validation cohorts, the sensitivity was 81.8% and 81.8%, respectively, and the specificity was 74.3% and 64.8%, respectively. The PPV was 40.9% and 22.0%, respectively, and the NPV was 94.9% and 96.7%, respectively.

**Table 4:** Diagnostic characteristics of the simplified risk score cut-off points in the three cohorts

	Derivation	Internal validation	External validation
Sensitivity % (95% CI)	83.3 (75.6–97.0)	81.8 (65.7–97.9)	81.8 (65.7–97.9)
Specificity % (95% CI)	76.2 (72.1–80.4)	74.3 (65.7–82.8)	64.8 (57.9–71.8)
Positive predictive value % (95% CI)	43.9 (36.4–51.3)	40.9 (26.4–55.4)	22.0 (13.0–30.9)
Negative predictive value % (95% CI)	93.4 (93.1–97.7)	94.9 (90.1–99.8)	96.7 (93.6–99.9)
Accuracy % (95% CI)	77.5 (73.9–81.2)	75.6 (68.0–83.2)	66.7 (60.2–73.1)

*CI* confidence interval

### Aetiologies of false-positive STEMI

The aetiology of the 134 cases of false-positive STEMI in all cohorts was classified. The distribution of the final diagnosis is shown in Table 5. Coronary spasm (28.4%) was the most common cause of false-positive STEMI. Other common aetiologies included primary rhythm disturbance (19.4%) and structural/valvular heart disease (12.7%). Causes (12.7%) other than cardiovascular events were subarachnoid cerebral haemorrhage, massive gastrointestinal bleeding, and metabolic causes. Among the total population, 15.7% were not diagnosed with any disease. In 134 patients with false-positive STEMI, emergency CAG was performed in half (67 patients), and coronary spasm (26, 38.8%) was the most common diagnosis, followed by no diagnosis (12, 17.9%), and primary rhythm disturbance (10, 14.9%).

**Table 5:** Aetiologies of false-positive STEMI

Final diagnosis, N (%)	Total	Patients with CAG
Coronary spasm	38 (28.4)	26 (38.8)
Primary rhythm disturbance	26 (19.4)	10 (14.9)
Structural/valvular heart disease	17 (12.7)	8 (11.9)
Myocarditis/pericarditis	8 (6.0)	5 (7.5)
Stress induced cardiomyopathy	4 (3.0)	2 (3.0)
Hypertensive emergency	2 (1.5)	0
Pulmonary embolism	1 (0.7)	0
Others	17 (12.7)	4 (6.0)
No diagnosis	21 (15.7)	12 (17.9)
Total	134	67

*STEMI* ST-segment elevation myocardial infarction, *CAG* coronary angiography

## Discussion

In this study, the overall incidence of false-positive STEMI was 16.3%. Age, characteristics of chest pain, reciprocal change on ECG, and concave morphology of ST elevation were independent predictors for false-positive STEMI and were included in the final prediction model. Our model was presented as a risk score that ranged from 0 to 8 points and showed a good level of accuracy for false-positive STEMI prediction, with an AUROC of 0.8 or higher. When the cut-off value of 2.5 was applied, high NPV and low PPV were obtained.

As noted, the overall incidence of false-positive STEMI was 16.3%, but there was a gap between the two hospitals (18.2% vs. 10.8%). Both hospitals used the same protocol, and the differences may have been due to differences in patient demographics; the patients in hospital B were younger than those in hospital A. The prevalence of false-positive STEMI has broadly been reported to be between 7.5% and 36.0% [8–14]. Not only patient characteristics but also study design can affect the incidence of falsely diagnosed STEMI. Studies that only included patients who received primary PCI showed a lower incidence of false-positive STEMI (7.5%–11.0%) [8, 10, 11, 16]. When calculating the incidence only in patients with primary PCI in our study, the incidence of false-positive STEMI was 9.4%. However, in a study that included all patients in whom the CP was activated for STEMI, the incidence increased to 36.0% [13]. The authors of that study applied a clinical scenario to patients who did not receive primary PCI for the judgment of false-positive STEMI, similar to our study. Most patients presenting with the typical characteristics of STEMI received primary PCI, and the proportion of false-positive STEMI patients in clinical scenario-applied patients was naturally higher (59.8% in our study) than that for primary PCI patients. Thus, the prevalence of false-positive STEMI may have been underestimated in studies that included only patients who underwent primary PCI.

Over-activation of the CP for patients with a high sensitivity for disease detection is reasonable to a certain degree, because not missing real STEMI is more important than false activation. Mixon et al. also noted this problem, stating that the rate of improper ECG patterns was 12.8% among patients who underwent CP activation in an investigation of the appropriateness of using ECG to evaluate STEMI [14]. In studies that included CP-activated cases as a denominator regardless of the appropriateness of ECG, the rate of false-positive STEMI was 25.6%–28.4%, higher than the actual value [14, 21]. In our study, we endeavoured to exclude these “false alarm” cases to clarify the precise study population. However, surprisingly, there were more patients than expected with ECG results that did not meet the ST-elevation criteria (331, 34.9%). This high incidence of false alarm could be influenced by government policy. In 2007, the Korean government began to grade hospitals according to the proportion of patients who successfully received primary PCI within 90 min of arrival, and the grade influences the hospital’s funding for STEMI patients. Therefore, physicians focus on activating the CP rather than on false alarms when they encounter unclear clinical situations in which STEMI is suspected.

Patients with new-onset LBBB were not included in our investigation. These patients tend to show a greater proportion of false-positive STEMI than ST-elevation on ECG, and Qiangjun et al. suggested a guideline using the Sgarbossa score to prevent unnecessary coronary intervention in patients with new-onset LBBB [22]. The ACC/AHA guideline insists that patients with new or presumably new LBBB should no longer be treated as STEMI-equivalent [23]. Thus, we devised our prediction model only for patients with ST-segment elevation, and if patients with new-onset LBBB were included, the incidence of false-positive STEMI would increase.

So far, only two previous studies have suggested a prediction model for false-positive STEMI [9, 16]. Eduardo et al.’s model only included somewhat static variables, such as patient demographics, risk factors, and underlying disease, and the area under the curve showed moderate predictive capacity (0.67) [9]. The risk score in Tonga et al.’s study included dynamic variables, such as chest pain and reciprocal change on ECG, similar to our study, and revealed good predictability, with an area under the curve of 0.88 [16]. We found that the ECG of false-positive STEMI had several differences from true STEMI: the height of the ST-elevation, location and number of involved leads, and incidence of concave morphology and reciprocal change. These ECG patterns for false-positive STEMI are similar to those reported in previous studies [8, 10], and concave morphology and reciprocal change were independent risk factors in our prediction model. STEMI is frequently accompanied by reciprocal changes, which have been suggested to distinguish STEMI from other diseases [24, 25]. Reciprocal change is also a surrogate marker for the severity of STEMI [24, 26]. Our risk score model showed a good ability to discriminate false-positive STEMI in the internal and external validation cohorts (AUROCs, 0.84 and 0.82, respectively). To our knowledge, this is the first study that attempted to validate a prediction model for false-positive STEMI with an external validation set.

Over the last decade, most EDs in tertiary hospitals have tried to achieve the goal of early door-to-balloon time with various strategies, such as a CP for primary PCI. However, Barnes et al. suggested that during their study period, reduced door-to-balloon time was accompanied by increased negative results of primary PCI [7]. Patients with false-positive STEMI have been reported to have relatively poor outcomes, because several foetal pathologic conditions such as aortic dissection, pulmonary thromboembolism, and cerebral haemorrhage that should be diagnosed without delay can show ST elevation on ECG [11, 12, 19, 27]. This is the time to focus on how we can reduce the incidence of negative primary PCI, rather than just speed up the procedure. In recently published studies, further decreases in door-to-balloon time did not improve patient mortality, suggesting that benefits from earlier PCI reached a point of diminishing return [15]. Until now, the decision of whether to perform primary PCI has depended on the clinical physician’s individual capacity. Our prediction model would help them make rapid decisions with better rationale. However, when the cut-off value was applied, the NPV of our risk model was 93.4%–96.7%, whereas the PPV was 22.0–43.9%. We propose that if patients who show ST elevation on ECG have a high score according to our risk model, they should also be assessed for other aetiologies. However, these assessments should be performed promptly, and preparations should be made to ensure immediate implementation of primary PCI in case of emergency. The most appropriate assessment tool is point-of-care ultrasonography, which has gradually become familiar to emergency physicians and is widely used in the emergency room [28]. Further research on the role of point-of-care ultrasonography use by emergency physicians to rapidly determine false-positive STEMI should be performed.

### Limitations

This study has several limitations. Firstly, it is possible that there were patients with appropriate ST elevation on ECG who died before CP activation. These patients may have been more likely to have STEMI, so the incidence of false-positive STEMI may have been reported to be higher than it actually was. Secondly, most of the patients who were included in this study are Koreans, and it may be difficult to apply the results to international patients. Thirdly, there were only 202 patients included in the external validation cohort, among which only 22 patients had false-positive STEMI. These numbers were not sufficient to test the accuracy of the model. Lastly, since our prediction model was analysed only retrospectively, it is necessary to evaluate its predictive power in future prospective studies.

## Conclusions

We presented a predictive model to help identify false-positive STEMI patients early in the ED. Our model should be investigated for accuracy through a more extensive validation study. Finally, prospective studies should be performed to determine whether our model will actually help reduce primary PCI for false-positive STEMI in a real clinical environment.

## Abbreviations

ACC/AHA: American college of cardiology/American heart association
AUROC: Area under the receiving operating characteristic curve
CAG: Coronary angiography
CI: Confidence interval
CP: Critical pathway
ECG: Electrocardiography
ED: Emergency department
LBBB: Left bundle branch block
MI: Myocardial infarction
NPV: Negative predictive value
NSTEMI: Non-ST-segment elevation myocardial infarction
OR: Odds ratio
PCI: Percutaneous intervention
PPV: Positive predictive value
STEMI: ST-segment elevation myocardial infarction
